# Chicago Pathological Society—Annual Meeting

**Published:** 1883-07

**Authors:** J. H. Tebbetts

**Affiliations:** Secretary


### ARTICLE XII.

CHICAGO PATHOLOGICAL SOCIETY. Annual Meetimg.

The society was called to order by the President, Dr. Lyman. It being the annual meeting, the first business of the evening was the election of officers for the ensuing year.

According to former custom of the society, an informal ballot was taken for president. Dr. Lyman having received twelve votes, the whole number, was unanimously elected. He, however, declined to serve, believing that rotation was the only way to prevent stagnation and loss of usefulness. He, however, offered the society his parlors for meeting, should no more central location present. On formal ballot, Dr. J. J. M. Angear received a majority of the votes, and his election was made unanimous by a *viva voce* vote. Dr. D. R. Brower was elected Vice-President, Dr. J. H. Tebbetts Sec'y and Treas. Censors, Dr. Wm. A. Walker and A. Lagorio.

Dr. Angear was then conducted to the chair, thanking the society for the honor it had conferred upon him, a comparative stranger to most of the members. He considered pathology as the foundation of practice; irregular physicians are never pathologists, or get up works on pathology. Pathology rests on anatomy, physiology and chemistry, and our practice consists in the application of these principles, on the laws of common sense.

Dr. Lyman rose to express his gratification at the present condition of the society, and suggested that, if possible, a permanent home be sought, at reasonable expense, where the society can cultivate its desire for pathological objects of interest and secure a cabinet of specimens. Dr. Lyman then moved that the officers of the society be appointed a committee to secure rooms in the neighborhood of the County Hospital, if the location be deemed sufficiently central. Carried.

In concluding, he repeated his kind offer, the use of his parlors for another year, if no better location present.

It was then moved by Dr. Bishop, after remarking the kind hospitality of Dr. Lyman, and his exertions, to which mainly the society owes its present flourishing condition, that a vote of thanks be tendered the retiring president for use of his parlors.

Unanimously carried.

The Secretary was then called on to make the Annual Report, which was read and approved, said report being ordered offered for publication in some journal, to exhibit our work and ambitions, and enlist the interest of those not yet members.

The Treasurer's Report was as follows :

DR.

April 25, 1882, to April 9, 1883 :

| | |
|---|---|
| To annual dues collected | $29 00 |

CR.

| | |
|---|---|
| By bills paid, printing, stationery, etc | $24 02 |
| By cash on hand | 4 98 |
| | $29 00 |

The following named gentlemen and ladies were proposed for membership :

Dr. C. A. Sanders, by Drs. Lyman and Dobbin.

Dr. Isabell R. Copp, by Drs. Mergler and Lyman.

Dr. W. A. Synon, by Drs. Bridge and Lyman.

Dr. Curtis was elected to membership.

Dr. G. Frank Lydston, before reading his paper, exhibited to the society an elixir of salicylate of iron, prepared by his brother, a pharmacist, from salicylic acid and tinct. ferri chloride. The qualities are antipyretic and antiseptic, and the preparation may be employed in all septic affections, in many cases as a substitute for quinia ; unlike tinct. ferri chlorid,, it does not check the secretions. In doses of ℥ ss it does not irritate the stomach, nor damage enamel of teeth, but is said, on authority of Drs. Brophy and Talbot, to act as a preservative, preventing fermentative action.

In his paper entitled "The Treatment of Varicocele," Dr. Lydston stated that, although not an uncommon affection, the annoyance was so great and the many forms of palliative treatment so worthless in checking growth and removing deformity, that some more effectual means of relief, if not cure, was much sought and very desirable. The indications for the operation are the real danger to life from hæmatocele, the great dospondency of patient, etc. The popular idea that fatal hæmorrhage may occur in the operation, and the large number who are treated by the various instrument makers, keep many interesting cases from ever being treated by the surgeon.

The various older methods of operation were then enumerated, and some of their imperfections touched upon.

We should seek a safe operation, although patient may have to retain the suspensory bandage, to complete the cure. The method more nearly followed by the author of the paper, was a modification, by Henry, of Sir Astley Cooper's operation, although not using same construction of clamp.

The sutures employed were " figure of eight " with chromated gut ligatures and silver pins—also used by Henry. The adhesive straps can best be dispensed with at first dressing, but advantageously used in granulating stage.

Oakum compresses, covered with borated cotton, are to be used, the edges of the parts dusted with iodoform, and a decalcified bone drainage tube inserted at most dependent portion.

In dressing the parts, he uses a hot sat. sol. of boracic acid, after the late Warren Greene. In healing, a first intention will often occur, but a granulating union is really preferable, as the thicker cicatrix forms a more solid and firm support.

A specimen of a scrotum removed in a case was then exhibited, in which the redundancy was enormous.

Five cases were then detailed, of which case V was perhaps the most interesting.

A hospital case—the man possessed the hæmorrhagic diathesis, and had, naturally, great fear of operation. Varicocele of left side, and of long standing. The usual symptoms, somewhat relieved by palliative treatment. Carbolized silk was used in absence of chromated gut, 5 pins, and other usual dressings. Union occurred by first intention; bleeding, however occurred on the third day, and wound had to be opened, clots exposed and turned out, when hæmorrhage checked without further trouble. Discharged from hospital in twenty days, cured. The after-treatment is always important, consisting of a good suspensory bandage, baths, laxatives, faradic current—all aid in restoring resiliency and tone to venous walls. The best bandage is, perhaps, the " U. S. Army and Navy," as it has a small pouch and is non-elastic, supporting the scrotum in lifting and walking.

In the subsequent discussion of the paper by the society, Dr. Lagorio mentioned a case, caused by masturbation, and treated by a bandage, potassum bromide, and moral suasion, in which a complete cure resulted. Hence the Doctor would, in recent cases, urge postponement of operation.

Thought many young men are troubled with slight varicocle, which might create considerable disturbance until the irritating cause be removed by marriage.

Dr. Bucher would inquire if horseback riding did not conduce to varicocele, as U. S. Government, during the Rebellion, had issued suspensory bandages to about one out of six horsemen, and every case was thus cured without operation. Thought a properly constructed saddle did not produce the trouble—for example, the McClellan and Mexican, which have an open "tree."

It was then moved and carried that a vote of thanks of the society be tendered Dr. Lydston, and his paper offered for publication.

The President suggested that the papers of the year be collected and published annually in pamphlet form.

Dr. Lyman suggested that if all papers could be published in one journal, a file of said journal would be our best collection of papers, and therefore moved that the CHICAGO MEDICAL JOURNAL AND EXAMINER be considered the medium of the society.

Unanimously carried.

Members present: Drs. Angear, Bishop, Bucher, Camp, Dobbin, Lagorio, Lydston, Lyman, Tagert, Treat, Tebbetts, Walker, and two visitors.

The society, on motion, adjourned.

J. H. TEBBETTS, Secretary.

